# The “new normal” osmotic threshold: Osmostat reset

**DOI:** 10.5414/CNCS110740

**Published:** 2022-01-10

**Authors:** Larissa G. Rigueto, Henrique M. Santiago, David J. Hadad, Antonio Carlos Seguro, Adriana Castello C. Girardi, Weverton M. Luchi

**Affiliations:** 1 Hospital Universitário Cassiano Antonio Moraes, Universidade Federal do Espírito Santo (HUCAM-UFES), Vitória, ES, and; 2 Universidade de São Paulo, Faculdade de Medicina, São Paulo, SP, Brasil; *Larissa G. Rigueto and Henrique M. Santiago contributed equally to this work.

**Keywords:** hyponatremia, inappropriate ADH syndrome, SIAD, osmostat reset

## Abstract

Hyponatremia is the most common electrolyte disorder in hospitalized patients. The syndrome of inappropriate antidiuresis (SIAD) is one of the leading causes of hyponatremia. Although not widely known, SIAD has a vast spectrum of etiologies and differential diagnoses and has been classically divided into four types (A, B, C, D). Frequently, when we use the term SIAD in clinical practice, it refers to subtype A, the so-called classic SIAD. The purpose of reporting this case is to make the clinicians aware of a specific subtype of SIAD, type C, an underdiagnosed entity called osmostat reset (OR). Due to similarities, OR often ends up being misinterpreted as classic SIAD. However, the differentiation between these two entities is crucial due to treatment implications. This manuscript highlights the use of an algorithm, based on the fraction of uric acid excretion, as an approach to the differential diagnosis of hyponatremia.

## Introduction 

Hyponatremia, defined as a plasma sodium (P_Na_) concentration of < 136 mEq/L, is the most common electrolyte disorder in clinical practice, affecting up to 30% of hospitalized patients. Although the syndrome of inappropriate antidiuresis (SIAD) is the leading cause of hyponatremia, differential diagnosis is wide and requires a systematic approach to define etiology and the correct therapy [1]. 

The most common pattern of SIAD, characterized by the erratic release of antidiuretic hormone (ADH) despite plasma osmolarity values (P_OSM_), is the classic syndrome of inappropriate ADH secretion (type A or classic SIAD). Among the diagnoses that might be misinterpreted as classic SIAD, due to similarities with euvolemic hypotonic hyponatremia in patients with normal renal and endocrine functions, there is an unrecognized condition called osmostat reset (OR). Unlike classic SIAD, in OR, there is a reduction in the osmotic threshold for ADH release, whereas the tubular capacity of urinary dilution and concentration are preserved [2].

Herein, we describe a case of hyponatremia in a 30-year-old man with co-infection of tuberculosis (TB) and HIV, initially diagnosed as classic SIAD but refractory to the usual management. Further investigation revealed that the hyponatremia was due to OR. Distinguishing between these two entities is clinically relevant because there are treatment implications. 

## Case report 

### Clinical history and laboratory tests 

A 30-year-old male patient with TB-HIV co-infection, who had abandoned treatment, was admitted with a history of daily fever, weight loss, and asthenia for ~ 2 months. He was diagnosed with disseminated TB with central nervous system (CNS) involvement. On physical examination, the patient was alert and oriented to person, place, and time. Blood pressure was normal, mucosas were hydrated, capillary refill time was below 3 seconds, and without peripheral edema. Initial laboratory results showed a P_Na_ concentration of 122 – 126 mEq/L with no neurological symptoms attributed to hyponatremia. His medical charts showed satisfactory diuresis, varying from 1,300 to 2,000 mL in 24 hours. 

### Further investigation 

Additional laboratory tests revealed preserved renal, thyroid, and adrenal functions. Blood glucose and triglycerides were ranging within the normal values. Serum protein electrophoresis did not detect any monoclonal hyperproteinemia, and a urinary sample analysis showed urinary sodium (U_Na_) of 191 mEq/L and a urinary osmolarity (U_OSM_) of 659 mOsm/L (Table 1). 

According to the data collected on physical exam and laboratory tests described in Table 1, the patient was diagnosed with euvolemic hypotonic hyponatremia. Due to the context of the underlying disease, the disorder was initially managed as classic SIAD. Despite the interventions with water restriction and a solute-rich diet (high sodium and protein concentration diet), there was no improvement in the P_Na_. 

Although clinical assessment of the volume status indicated a preserved extracellular fluid volume, a borderline low-normal systolic blood pressure (90 – 100 mmHg) and a slightly elevated plasma renin activity (Table 1) suggested a possible hypovolemic state. Since overlaps in the laboratory findings between SIAD and renal salt wasting (RSW) are common, and recognizing that the assessment of volume status is challenging in critically ill patients with significant weight loss, we chose to initiate volume expansion with intravenous infusion of sodium chloride 0.9% (85 mL/h for 12 hours, 1 L total). P_Na_ concentrations, however, did not increase, which goes against RSW (Table 1). 

The investigation continued by requesting a plasma uric acid concentration (P_AU_) and calculating the fractional excretion of urate (FE_URATE_), the results of which were 7.8 mg/dL and 4.5%, respectively. In addition, significant U_OSM_ fluctuations were observed throughout hospitalization (Table 1). These findings do not match the typical hypouricemia with elevated FE_URATE_ and “fixed” U_OSM_ values commonly seen in classic SIAD. 

**Figure 1.**
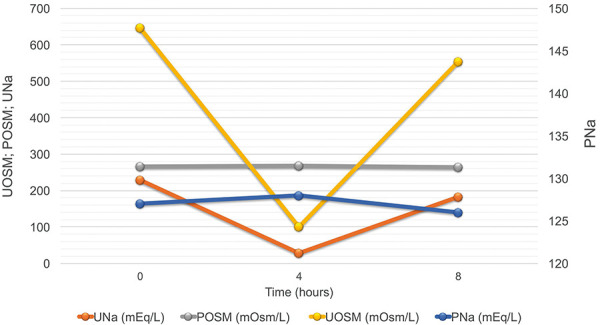
Water load test. The patient underwent an intravenous infusion of 15 mL/kg of electrolyte-free water (glucose 5%) for 1 hour on day 20 of hospitalization. There was a significant drop (> 80%) in urinary osmolarity (U_OSM_) from T0 (646 mOsm/L) to T4 (100 mOsm/L), with no oscillation in plasma sodium concentration (P_Na_ at T0 = 127, T4 = 128 mEq/L), indicating preserved urinary dilution capacity, confirming the osmostat reset diagnosis. U_Na_ = urinary sodium concentration; P_OSM_ = plasma osmolarity.

In this scenario, classic SIAD and RSW were considered improbable, and given the normal P_AU_ and FE_URATE_ values, the RO hypothesis became plausible. Subsequently, the patient underwent a fluid load test consisting of an intravenous infusion of 15 mL/kg of 5% glucose for 30 minutes, during fasting, and collection of serum and urinary samples at 0, 4, and 8 hours after the start of the infusion (Figure 1). The laboratory analysis showed a preserved urinary dilution capacity. A reduction of 83% in U_OSM_ was obtained from T0 to T4, without any significant P_Na_ change, confirming the OR diagnosis and ruling out the classic SIAD hypothesis. 

### Diagnosis 

Hypotonic hyponatremia with normal extracellular volume (euvolemic) secondary to OR in a patient with TB-HIV co-infection. 

### Follow-up 

Specific approaches for hyponatremia were suspended, and TB treatment continued. The patient had a partial improvement in P_Na_ concentration, being discharged from the hospital with a P_Na_ of 132 mEq/L. 

## Discussion 

Unlike classic SIAD (type A), described by Schwartz and Bartter in 1967, in which there is an abnormal release of ADH independent of P_OSM_, hyponatremia in OR is related to a downward reset of the osmotic threshold for ADH release and thirst stimulation. The term OR was first introduced in 1976 by DeFronzo et al. [4] to describe a scenario of euvolemic hypotonic hyponatremia in 4 patients, 3 of them with pulmonary TB, refractory to therapy with hypertonic saline, and whose water load test demonstrated preserved capacity for urinary dilution. Later, OR was classified as a new subtype of SIAD, type C, even though some authors disagree and consider it a separate entity [3, 4]. 

The stimulus for ADH release is triggered by an increased P_OSM_, which is detected by hypothalamic osmoreceptors (osmostat). Baroreceptors at the aortic arch and carotid sinus also stimulate ADH release when hypovolemia is present. The typical threshold for ADH release is set within a narrow range of 275 to 295 mOsm/L. However, in OR, there is no abnormal/excessive ADH secretion; instead, ADH release is linked to a “new normal” osmotic and thirst threshold, which is shifted to lower levels [2]. Thus, if we assume that in OR these new values for threshold are fixed between 265 and 285 mOsm/L, a patient with a P_OSM_ of 280 mOsm/L who typically would have virtually suppressed ADH levels ends up having elevated ADH levels and experiencing thirst even though he or she is hyponatremic. 

One of the mechanisms proposed to explain OR is related to a primary disturbance of osmoreceptor neuronal cells known as “sick cell syndrome”, triggered by metabolic or nutritional cell dysfunctions and alterations of the plasma membrane permeability. These cellular dysfunctions would be responsible for the loss of non-diffusible and diffusible intracellular solutes, which would lead to “cellular dehydration” and stimulation of ADH secretion. It has been hypothesized that these changes may also be present in patients with chronic hyponatremia, functioning as an adaptive cellular process to the chronic drop in P_OSM_. Another proposed mechanism would result from the interruption of inhibitory pathways through baroreceptors, either by autonomic neuropathy or by carcinomatous invasion. Disruption of these pathways would lead to false signs of effective arterial volume depletion and subsequently ADH release at lower P_OSM_ levels [5, 6]. 

According to some authors, OR represents ~ 33% of all 4 SIAD subtypes [6]. Among the associated clinical conditions, infection by *Mycobacterium tuberculosis* stands out [7]. In a previous study, ~ 32% of cases of hyponatremia in patients with pulmonary/miliary TB were attributed to OR. Other conditions include pregnancy, old age, quadriplegia, psychosis, cerebral hemorrhage, encephalitis, midline defects (cleft lip and palate), agenesis of the corpus callosum, hypothalamic cyst, dementia (Lewy bodies), alcoholism, malnutrition, malignancy (stomach, colon, small cell lung carcinoma), and pneumonia by *Pneumocystis carinii* [2, 8]. 

Clinical suspicion of OR should be considered in patients with mild/moderate hyponatremia whose presumed diagnosis is classic SIAD, or even RSW, but with low P_Na_ levels maintained stable, without significant elevations, despite the institution of therapeutic approaches, such as water restriction and increased supply of solutes in the case of classic SIAD, or the infusion of sodium chloride 0.9% in the RSW. Furthermore, unlike the classic SIAD, where the U_OSM_ presents relatively “fixed” values, regardless of the administration of hypotonic or hypertonic solutions, in OR, there are significant oscillations in the U_OSM_, as the capacity for dilution and urinary concentration remains preserved [2, 4, 9]. Table 2 summarizes the main differences between classic SIAD and RO [5, 9, 10, 11]. 

**Figure 2.**
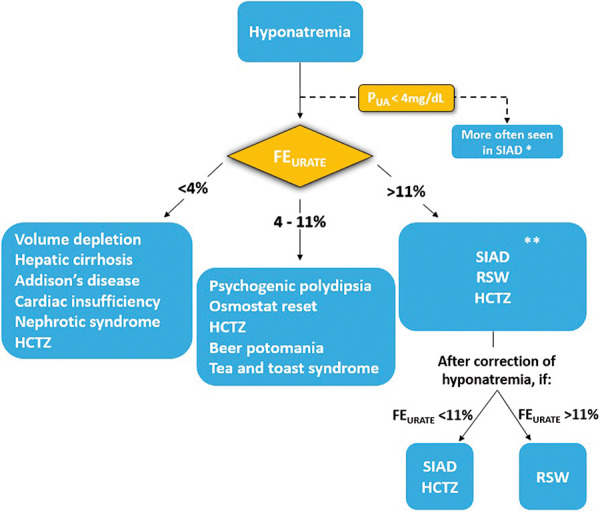
Use of FE_URATE_ as a tool in the differential diagnosis of hyponatremia. Adapted from Imbriano et al. [12]. FE_URATE_ can be obtained by calculating the product of urinary urate and serum creatinine, divided by the product between plasma uric acid and urinary creatinine, multiplied by 100 (FE_URATE_ = U_URATE_ X P_CR_ / P_AU_ X U_CR_ × 100). *The presence of hypouricemia (< 4 mg/dL) is more suggestive of SIAD, but it may also be present in cases of RSW and thiazide-induced hyponatremia. **In cases of hyponatremia in which SIAD and thiazide are the most probable differential diagnosis, Fenske et al. demonstrated that an FE_URATE_ cut off > 12% had a positive predictive value of 100% in defining SIAD as the cause factor [13]. HCTZ = hydrochlorothiazide; SIAD = syndrome of inappropriate antidiuresis; RSW = renal salt wasting; FE_URATE_ = fractional excretion of urate; P_UA_ = plasma uric acid concentration.

An important clue for the diagnosis of OR is the presence of normal P_AU_ and FE_URATE_, since cases of classic SIAD and RSW typically have a low concentration of P_AU_ (< 4 mg/dL) and a high FE_URATE_ (> 11%). Traditionally, the differential diagnosis of hyponatremia includes the classification of volume status; however, it is well known that the assessment of extracellular volume by clinical signs has limited sensitivity and specificity. In this context, the FE_URATE_ calculation can be a valuable tool to aid in the diagnostic definition, as illustrated in Figure 2 [12, 13]. 

The definitive diagnosis of OR is stated by performing a fluid load test, which consists of the administration of water orally or 5% glucose intravenously in a volume of 10 – 20 mL/kg for 30 minutes associated with serial sample collection serum and urinary tests for the evaluation of P_Na_ and U_OSM_. Diagnostic confirmation occurs when the urinary dilution capacity is preserved, observed by a drop in urinary osmolarity > 80% compared to the initial value (from T0 to T4h), as shown in Figure 1 [8, 10]. 

The distinction between classic SIAD and OR is clinically relevant because they are entities that, even within the broad spectrum of inappropriate ADH secretion, are pathophysiologically different, thus requiring specific treatment approaches. Osmostat reset hyponatremia does not respond to the conventional clinical management of classic SIAD. When OR occurs because of a reversible pathology, as described in this case, the osmostat often returns to normal after treatment of the underlying disease and does not require specific therapy attempting to correct serum sodium concentration [2, 8]. 

## Funding 

The authors received no financial support for the research, authorship, and/or publication of this article. 

## Conflict of interest 

The authors declared no potential conflict of interest with respect to the research, authorship, and/or publication of this article. 

**Table 1.** Laboratory parameters.

Blood	Admission	Day 5	Day 10*	Reference range
Creatinine (mg/dL)	0.65	0.66	0.63	0.7 – 1.2
Urea (mg/dL)	32	35	32	10 – 50
Potassium (mEq/L)	4.0	4.2	4.3	3.5 – 5.1
Sodium (mEq/L)	124	121	123	135 – 145
Glucose (mg/dL)	112	135		70 – 99
Uric acid (mg/dL)	7.8		4.7	3.9 – 8.1
TSH (UI/mL)	2.1			0.35 – 4.94
T4 free (ng/dL)	0.85			0.7 – 1.48
Cortisol at 8h (μg/dL)		17.3		3.7 – 19.4
Renin activity time (ng/mL/h)		5.1		0.2 – 3.3
Plasma osmolarity (mOsm/L)	260	256		275 – 295
Urine (spot urine)				
Sodium (mEq/L)	191	69	184	40-220
Urinary osmolarity (mOsm/L)	659	183	478	
FE_UR_ (%)	42%			
FE_URATE_ (%)	5.0		9.0	4 – 11
Diuresis				
24-hours urine collection (mL)	1,700	2,000	2,300	

*D10: after infusion of 1,000 mL of 0.9% NaCl. FE_UR_ = fractional excretion of urea; FE_URATE_ = fractional excretion of urate.

**Table 2.** Main clinical and laboratory findings that allow the differentiation between classic SIAD (type A) and osmostat reset (SIAD type C).

Clinical and laboratory findings	Classic SIAD	Osmostat reset
Hypotonic hyponatremia (P_OSM_ < 275 mOsm/L)	Yes	Yes
Euvolemia	Yes	Yes
U_OSM_ > 100 mOsm/L	Yes	Yes
Significant variations in U_OSM_	No (U_OSM_ “fixed”)	Yes
U_Na_ > 30 mEq/L	Yes	Yes
Normal renal, thyroid, and adrenal functions	Yes	Yes
No recent use of diuretics	Yes	Yes
P_AU_ < 4 mg/dL	Common	No
FE_AU_ > 11%	Yes	No
Plasma urea levels	Low-normal (< 30 mg/dL)	Normal
FE_UREA_	> 55%	< 55%
Worsen of hyponatremia due to sodium chloride 0.9% intravenous infusion	Yes	No
Improvement of hyponatremia due to water restriction and high solute diet	Yes	No
Inappropriately high ADH and copeptin levels regarding plasma osmolarity	Yes	Yes
Response to water load test	< 80%	> 80%*

*Usually reaches values < 100 mOsm/L.

## References

[1] Upadhyay A Jaber BL Madias NE Epidemiology of hyponatremia. Semin Nephrol. 2009; 29: 227–238. 19523571 10.1016/j.semnephrol.2009.03.004

[2] Kuthiah N Er C Reset Osmostat: A challenging case of hyponatremia. Case Rep Med. 2018; 2018:5670671. 30532786 10.1155/2018/5670671 PMC6247647

[3] Robertson GL Regulation of arginine vasopressin in the syndrome of inappropriate antidiuresis. Am J Med. 2006; 119: S36–S42. 16843083 10.1016/j.amjmed.2006.05.006

[4] DeFronzo RA Goldberg M Agus ZS Normal diluting capacity in hyponatremic patients. Reset osmostat or a variant of the syndrome of inappropriate antidiuretic hormone secretion. Ann Intern Med. 1976; 84: 538–542. 1275354 10.7326/0003-4819-84-5-538

[5] Flear CT Singh CM Hyponatraemia and sick cells. Br J Anaesth. 1973; 45: 976–994. 4127997 10.1093/bja/45.9.976

[6] Zerbe R Stropes L Robertson G Vasopressin function in the syndrome of inappropriate antidiuresis. Annu Rev Med. 1980; 31: 315–327. 6772090 10.1146/annurev.me.31.020180.001531

[7] Hill AR Uribarri J Mann J Berl T Altered water metabolism in tuberculosis: role of vasopressin. Am J Med. 1990; 88: 357–364. 2327423 10.1016/0002-9343(90)90489-z

[8] Feder J Gomez JM Serra-Aguirre F Musso CG Reset Osmostat: Facts and Controversies. Indian J Nephrol. 2019; 29: 232–234. 31423055 10.4103/ijn.IJN_307_17 PMC6668321

[9] Decaux G Musch W Clinical laboratory evaluation of the syndrome of inappropriate secretion of antidiuretic hormone. Clin J Am Soc Nephrol. 2008; 3: 1175–1184. 18434618 10.2215/CJN.04431007

[10] Hoorn EJ van der Lubbe N Zietse R SIADH and hyponatraemia: why does it matter? NDT Plus. 2009; 2: iii5–iii11. 19881934 10.1093/ndtplus/sfp153 PMC2762826

[11] Filippatos TD Liamis G Christopoulou F Elisaf MS Ten common pitfalls in the evaluation of patients with hyponatremia. Eur J Intern Med. 2016; 29: 22–25. 26706473 10.1016/j.ejim.2015.11.022

[12] Imbriano LJ Mattana J Drakakis J Maesaka JK Identifying Different Causes of Hyponatremia With Fractional Excretion of Uric Acid. Am J Med Sci. 2016; 352: 385–390. 27776720 10.1016/j.amjms.2016.05.035

[13] Fenske W Störk S Koschker AC Blechschmidt A Lorenz D Wortmann S Allolio B Value of fractional uric acid excretion in differential diagnosis of hyponatremic patients on diuretics. J Clin Endocrinol Metab. 2008; 93: 2991–2997. 18477658 10.1210/jc.2008-0330

